# Supercritical CO_2_ Extraction and Tandem Mass Spectrometry of the Medicinal Plant Sagan Dalya (*Rhododendron adamsii*)

**DOI:** 10.3390/ph18121823

**Published:** 2025-11-28

**Authors:** Mayya P. Razgonova, Alexander M. Zakharenko, Kirill S. Golokhvast

**Affiliations:** 1 N.I. Vavilov All-Russian Institute of Plant Genetic Resources, 42-44 Bolshaya Morskaya Street, 190000 Saint-Petersburg, Russia; 2 Far Eastern Federal University, 8 Sukhanova Street, 690950 Vladivostok, Russia; 3 Higher Engineering School “Agrobiotek”, National Research Tomsk State University, Lenin Ave, 36, 634050 Tomsk, Russia; rarf@yandex.ru (A.M.Z.); golokhvast@sfsca.ru (K.S.G.); 4 Siberian Federal Scientific Centre of Agrobiotechnology RAS, 7 Centralnaya Street, 633501 Krasnoobsk, Russia

**Keywords:** *Rhododendron adamsii*, Sagan Dalya, supercritical fluid extraction, tandem mass spectrometry, phenolic compounds, daurichromenic acid, cannabigerorcinic acid

## Abstract

**Background:** In Siberian folk medicine, Sagan-Dalya (*Rhododendron adamsii* Rehder) of the *Ericaceae* family is used as a tonic and restorative in the form of infusions and decoctions. Pharmacological studies have shown that alcoholic extracts of this plant enhance performance and have anti-inflammatory and immunomodulatory effects. *Rhododendron adamsii* shoots accumulate essential oil (up to 1.6%), flavonoids (1.8–3.0%), tannins (up to 6.9%), phenolic carbolic acids, β-sitosterin, oleanolic and ursolic acids, simple phenolic compounds, and coumarins. **Methods:** Supercritical carbon dioxide extraction (SC-CO_2_) is the most preferred environmentally friendly and selective method for extracting these natural compounds from the plant matrix of *Rh. adamsii* due to their high thermolability. Tandem mass spectrometry was applied to detect chemical compounds. Mass-spectrometry (MS) analysis was performed on an ion trap equipped with an ESI source in negative and positive ion modes. The capture rate was one spectrum/s for MS and two spectrum/s for MS/MS. All experiments were repeated three times. A four-stage ion separation mode (MS/MS mode) was implemented. **Results:** The operative parameters and working conditions have been optimized by different pressure (100–400 bars) and temperature (31–70 °C) regimes, and CO_2_ flow rate (10–25 mL/min) with 1 C_2_H_5_OH as a co-solvent. The extraction time varied from 60 to 90 min. The maximum global yield of biologically active substances (BAS) from *R. adamsii* leaves and stems was observed under the following extraction conditions: Pressure: 350 bar, extraction temperature: 65 °C, extraction time: 1 h; the global yield of BAS was 8.5 mg/g of plant sample; the share of the co-solvent (C_2_H_5_OH) was 2%. In total, forty-nine different BAS were identified in the *Rh. adamsii* SC-CO_2_ extracts. **Conclusions:** The obtained results may shed new light on the scientific basis for the traditional medicinal use of *Rh. adamsii* leaf and stem extracts. The pharmacological contribution of the identified phytocannabinoids requires further detailed study. It is hypothesized that the excellent transdermal permeability of supercritical extracts may open new therapeutic approaches using transdermal formulations based on SC-CO_2_ extracts of *Rh. adamsii*.

## 1. Introduction

The genus *Rhododendron* is one of the largest genera of the *Ericaceae* family, totaling about 800 species [1]. The taxonomy of the genus *Rhododendron* is complex and not yet sufficiently elaborate. The large size of the genus explains why there is no single classification of its taxa. The system developed by Russian scientists divides the genus *Rhododendron* into subgenera and series. In its *Rhododendron adamsii*, Rehder assigned it to the subgenus *Osmothamnus* Maxim. (series *Fragrantia* E. Busch) [2,3].

A detailed study of plant metabolites is an extremely important task, because it allows us to identify chemicals (or complexes of substances) that determine certain beneficial properties of plants. On the territory of Siberia and the Far East, a huge number of very interesting plants grow, the beneficial properties of which have long been used in traditional medicine [4,5]. At the same time, from a chemical point of view, many of these plants have been studied only superficially, although the need to obtain comprehensive information on the composition of secondary metabolites is obvious, and, in particular, this applies to those plants that have long been used in folk medicine.

**Figure 1:**
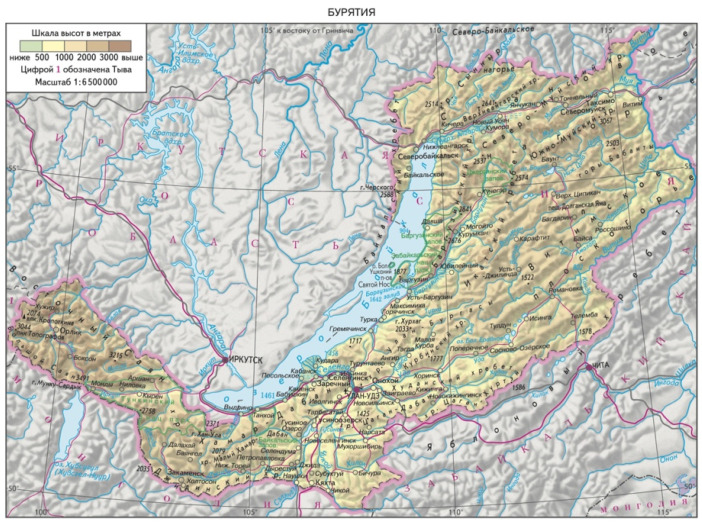
Territory of Buryatia (dissipating area of *Rh. adamsii* Rehder).

**Figure 2:**
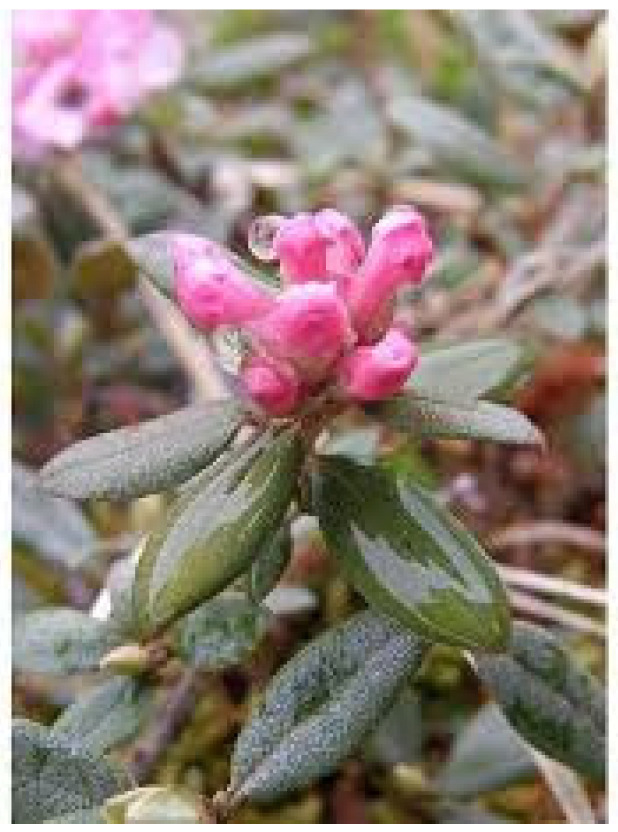
*Rh. adamsii* Rehder (Sagan Dalya).

A very interesting, rarely studied medicinal plant, widespread in the temperate climate zone on the border with Mongolia, is *Rh. adamsii* Rehder. This medicinal plant is widely distributed in Buryatia, the area around Lake Baikal, Mongolia, and Northeast China (Figure 1 and Figure 2). Traditional medicine uses different types of rhododendrons to treat a number of diseases of the respiratory system, the gastrointestinal tract, chronic skin diseases, hypertension, rheumatism, helminth infections, and others [6]. A very pungent odor is produced when burning *Rh. adamsii* leaves, caused by the rapid evaporation of volatile terpenes and other biologically active compounds. Treatment with *Rh. adamsii* smoke, as well as fumigation, has long been used by the indigenous peoples of Siberia, Buryatia, and Mongolia as a psychoactive and analgesic remedy, as well as for a variety of ailments related to the lungs and bronchi [7,8,9].

The essential oil obtained from *Rh. adamsii* is rich in volatile compounds, with many compounds such as isoledene, aromadendrene, humulene, α- and β-pinenes, β-myrcene, cis-β-ocimene, β-farnesol, spathulenol, β-elemenone, germacrone, γ-murolene, β-selinene, ledene, α-farnesol, β-cadinene, trans-nerolidol, being present in both the leaves and stems of the plant [7,8]. In almost all samples of essential oil from the leaves and stems of *Rh. adamsii*, the chemical compound 4-phenyl-2-butanone was found, the content of which ranges from 3 to 13%, as well as the chemical compound 4-phenyl-2-butanol, the content of which ranges from 1.9 to 7.4% [7,8,9].

The quantitative content of the components of the essential oil of the leaves and stems of plant samples collected in different years varies greatly. It has also been noted that changes in the chemical composition of the essential oils are observed during the storage of the raw materials. The following components predominate in the composition of the essential oil of *Rh. adamsii* (in descending order): germacrone (26.2%), ± trans-nerolidol (18.4%), seline-3,7(11)-diene (8.3%), guaia-3,9-diene (5.8%), (+)-ledol (4.6%), phenyl methyl ketone (3.9%), (+)-ledene (2.5%), 1H-cyclopro[e]azulene, and decahydro-1,1,7-trimethyl-4-methylene (2.4%).

Almost all acids with a number of carbon atoms from 12 to 30 are present in free form in the leaves and stems of *Rh. adamsii*, including odd-numbered, *inter alia* iso-fatty acids [7,8,9,10]. The proportion of free acids from the mass of leaves is 3.6%, and in the stems is 1.2%. The total content of fatty acids in the leaves is 6.2%, and in the stems, 2.4% of the mass of raw materials. The highest relative content is behenic acid (1.9%) in its free form in the leaves of *Rh. adamsii*. The content of other acids is in the range of 0.1–1.0%. Of the free and esterified acids, linoleic acid is the major component in stems. Together with linolenic acid, it makes up the bulk of all the acids of the stems of the plant. In addition to these, it is possible to note an increased content of acids with an even number of carbon atoms from C_16_ to C_24_; the rest is 1% or less [9,10].

Supercritical fluid extraction (SFE) and supercritical liquid chromatography (SFC) have been used since the late 1970s for food analysis and for determining the lipid content of food and levels of toxicants. The use of SFC for fractionation (supercritical fluid fractionation, SFF) and/or enrichment of certain components in products has been reported since the 1980s. Extracts obtained using SC-CO_2_ contain, in general, all biologically active components along with inert mixtures of extracted compositions [11].

Supercritical CO_2_ extraction, which occurs when using high pressures, is an excellent technique for producing natural thermolabile compounds. Furthermore, the extraction products do not contain residual organic solvents, which is typical of traditional extraction methods. The use of supercritical extraction of various plant matrices also offers the following advantages: easy removal of solvent from the resulting extract, high selectivity, and the use of moderate temperatures during the extraction process, which allows for the preservation of particularly valuable heat-labile compounds in extracts of various medicinal plants [12,13,14]. The results of SC-CO_2_ extraction of rhododendron leaves and branches, in particular *Rhododendron tomentosum* Harmaja, indicate that when using this technology, the extract contained all the BAS of the plant, as well as inert mixtures of extracted compounds [15].

Here, we studied the *Rh. adamsii* Rehder leaf and stem extracts and report BAS within the extracts. Through this work, we intended to clarify several points of the chemical composition, as well as the possibility and effectiveness of SC-CO_2_ extraction of BAS from stems and leaves of *Rh. adamsii*.

## 2. Results

**Figure 3:**
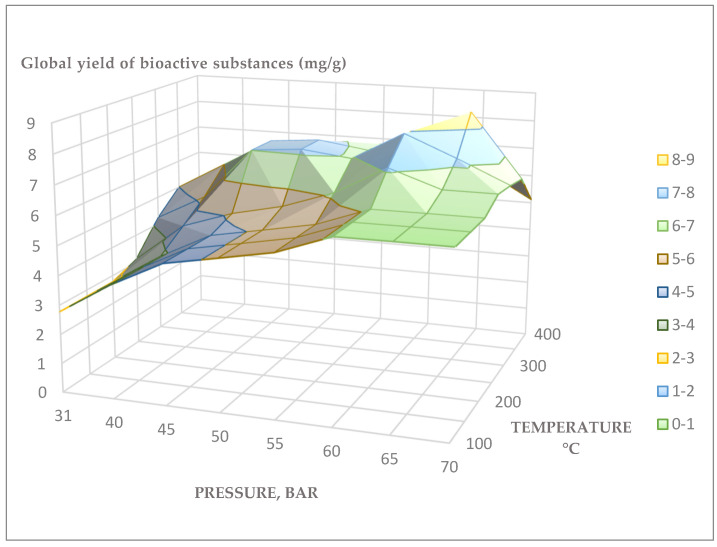
Three-dimensional graph of the global yield of BAS during SC-CO_2_ extraction of the *Rh. adamsii* leaves and stems.

**Figure 4:**
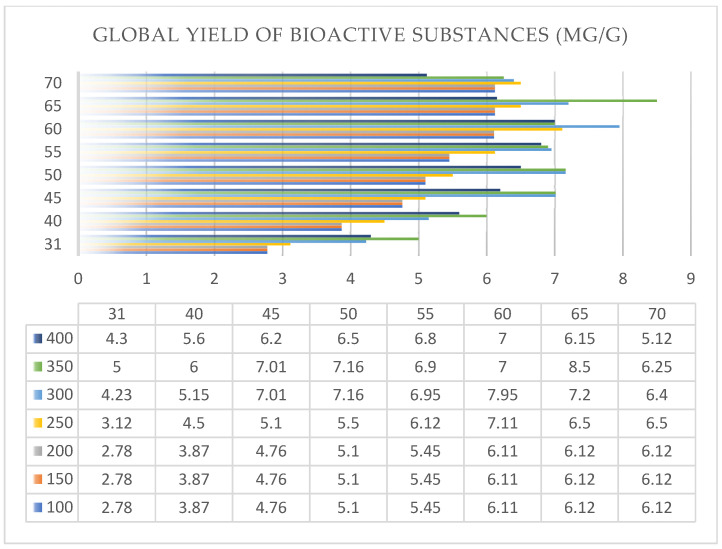
The graph of the global yield of BAS during SC-CO_2_ extraction of the *Rh. adamsii* leaves and stems, and the data distribution table.

*Rhododendron adamsii* leaves and stems were extracted with SC-CO_2_ at varying pressure and temperature regimes (Table A1). To increase the extraction efficiency (increase in solvent polarity), co-solvent (C_2_H_5_OH) was used in an amount of 1% of the total solvent. The quantitative ratio of the *Rh. adamsii* extract obtained by supercritical extraction was achieved by evaporating the CO_2_ extract and calculating the ratio of the mass of the extracted *Rh. adamsii* leaves and stems to the dry mass of the obtained extract. Below is a three-dimensional graph of the global yield of BAS during SC-CO_2_ extraction of the *Rh. adamsii* leaves (Figure 3 and Figure 4).

The supercritical pressures applied ranged from 100 to 400 bar, and the extraction temperature ranged from 31 to 70 °C. The co-solvent (C_2_H_5_OH) was used in an amount of 1% of the total solvent amount.

The maximum global yield of BAS from *Rh. adamsii* leaves and stems were observed under the following extraction conditions:

- Supercritical pressure: 300 bar, extraction temperature: 60 °C, extraction time: 1 h; the global yield of BAS was 7.95 mg/g of plant sample; the share of the co-solvent (C_2_H_5_OH) was 2%;
- Supercritical pressure: 350 bar, extraction temperature: 65 °C, extraction time: 1 h; the global yield of BAS was 8.5 mg/g of plant sample; the share of the co-solvent (C_2_H_5_OH) was 2%.

**Figure 5:**
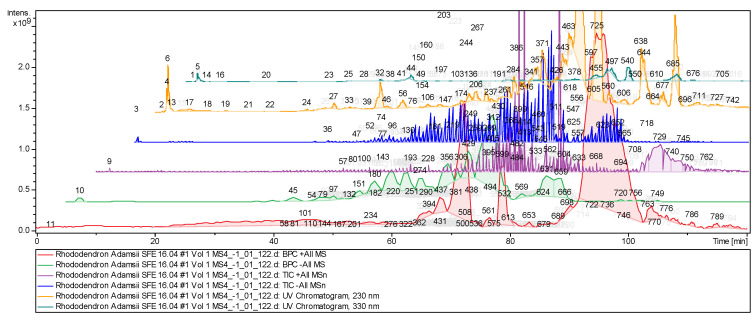
Chemical profiles of the *Rh. adamsii* sample represented an ion chromatogram from supercritical CO_2_-exract (red line—positive ion signal intensity graph; green line—negative ion signal intensity graph; violet line—total positive ion intensity, blue line—total negative ion intensity).

High-accuracy mass spectrometric data were recorded on an ion trap amaZon SL (BRUKER DALTONIKS, Bremen, Germany) equipped with an ESI source in the mode of negative and positive ions. The capture rate was one spectrum/s for MS and two spectrum/s for MS/MS. Under these conditions, a total of 800 peaks were detected in the ion chromatogram (Figure 5).

A unifying system table was compiled of the molecular masses of the target analytes isolated from the supercritical CO_2_ extract of *Rh. adamsii* for ease of identification (Appendix A Table A1). Among them, fifty chemical compounds are characteristic of *Rh. adamsii* species were tentatively identified in the CO_2_ extract. The chemical constituents were identified by comparing their mass spectra, retention index, and mass spectrometry fragmentation with a home-library database built by the Group of Biotechnology, Bioengineering and Food Systems at the Far-Eastern Federal University, based on data from other spectroscopic techniques, such as nuclear magnetic resonance, ultraviolet spectroscopy, and MS, as well as data from the literature that is continually updated and revised.

## 3. Discussion

Nowadays, due to the negative impact of many types of industrial extraction on the environment, the concept of green extraction has been introduced to ensure the protection of both the environment and consumers. The concept of green extraction also directly affects the growing competition between industries to be more environmentally friendly (use of by-products, biodegradability) and innovative [16]. According to this approach to green extraction, non-traditional extraction methods are used for the actual separation of bioactive compounds, for example, methods based on the use of compressed fluids as extractants, such as subcritical water extraction (SWE), SFE, pressurized fluid extraction (PFE), or accelerated solvent extraction (ASE) [17,18].

Supercritical extraction is rightfully referred to as a “green” extraction method and can be used as an alternative in comparison with other traditional extraction methods [19,20]. Supercritical fluid extraction has also been proposed as an alternative to purification procedures for obtaining extracts enriched in specific compounds of interest; this applies, for example, to wheat germ oil, green coffee oil obtained by pressing, rice bran oil, or crude palm oil [21,22].

The SFE of soy isoflavones has also been extensively studied; aqueous methanol appears to be the most suitable modifier for SC-CO_2_ extraction of isoflavones, although the use of acetonitrile has also been tested. Daidzein and genistein (sourced from soy products) have been successfully extracted at high pressures of 350 to 500 bar [22]. These compounds, as well as other bioactive substances, were extracted using SFE from a number of plant matrices: stilbenes such as cajanin-stilbene (a group of highly durable and chemically stable stilbene-derived dyes used for cellulose fiber) from *Cajanus cajan* (pigeon pea), cinnamon derivatives from propolis, or carotenoids and flavonoids from black rice [23,24,25].

The SFE has proven to be a useful tool for studying the fatty acid profile of fish oil. Fractionation of fish oil with SC-CO_2_ to obtain omega-3-enriched fractions was possible at various temperatures and pressures. Tuning the extraction parameters was found to make supercritical extraction a useful procedure for effectively modifying the lipid composition, resulting in a high-value functional product. One of the few studies investigating SFE using a solvent other than CO_2_ concerns the extraction of fatty acids from a fish oil sample. In this case, the potential of ethane as an alternative to the more common use of CO_2_ for fatty acid extraction was assessed. Furthermore, thermodynamic modeling was used to obtain the most suitable conditions for extracting the maximum possible amounts of eicosapentaenoic (EPA) and docosahexaenoic (DHA) acids. Ethane provides superior selectivity and higher solubility for EPA and DHA esters compared to other esters containing the same number of carbon atoms. At 60 °C and 84 bar, a concentration of 60% fatty acid ethyl esters can be obtained using ethane as a supercritical solvent [26,27].

Phospholipids are widely used in the food industry, not only for their technological properties but also for their bioactive capacity. Two SFE processes (without and with ethanol as a modifier) in combination with a supercritical antisolvent procedure have been proposed as an effective method for obtaining highly pure phospholipid extract from egg yolk powder. The SFE is also effective for more than fivefold concentration of phospholipids present in whey and buttermilk powders [27,28]. Thus, the use of SC-CO_2_ extraction is an effective scientific method for extracting biologically active compounds, in particular for extracting bioactive compounds from extracts of *Rh. adamsii* leaves and stems.

**Figure 6:**
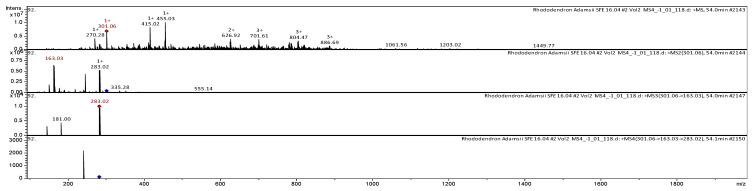
CID-spectrum of rhamnocitrin from CO_2_-extracts of *Rh. adamsii*, *m*/*z* 303.09.

The polyphenolic compounds detected in our study were further classified as flavonols, flavones, flavanones, coumarins, lignans, phenolic acids, and anthocyanidins. Compounds of other chemical groups included organic acids, polysaccharides, omega-3 fatty acids, triterpenoids, monobasic saturated carboxylic acids, pterocarpans, phytocannabinoids, cyclohexanecarboxylic acids, and others. Overall, the metabolites detected in our study belonged to 16 classes of chemical compounds. The largest number of polyphenolic compounds was flavonols (9), followed by coumarins (4) and phenolic acids (4). Our results indicate that twenty-one compounds of the polyphenol group were tentatively identified in the extracts of *Rh. adamsii* leaves and stems (Appendix A Table A1). The collision-induced dissociation spectrum (CID-spectrum) in negative ion modes of rhamnocitrin from CO_2_-extracts of *Rh. adamsii* is shown in Figure 6.

The [M+H]^+^ ion produced two fragment ions with *m*/*z* 283.02 and *m*/*z* 163.03 (Figure 6). The methyl radical loss: a clear peak at *m*/*z* 283.02 confirms the presence of the methoxy group (C_6_H_12_O_6_ + H -H_2_O (18 Da) = C_6_H_11_O_5_ (*m*/*z* 163.03).

C_9_H_9_O_4_ (*m*/*z* 181.00) is the key ion. It indicates a methoxy-hydroxy (7-OCH_3_, 5-OH) A-ring. 10 Da heavier than kaempferol’s A-ring. It was identified as rhamnocitrin in the mass spectrometric bibliography in extracts of *Mentha* [29]; *Astragali radix* [30]; *Lonicera caerulea* [31]; *Phyllanthus urinaria* L. [32].

**Figure 7:**
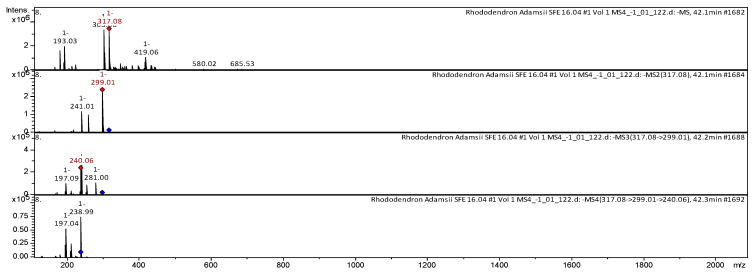
CID-spectrum of myricetin from CO_2_-extracts of *Rh. adamsii*, *m*/*z* 317.08.

The CID-spectrum in negative ion modes of myricetin from CO_2_-extracts of *Rh. adamsii* is shown in Figure 7.

The [M-H]^−^ ion produced one fragment with *m*/*z* 317.08 (Figure 7). The fragment ion with *m*/*z* 317.08 yields two daughter ions with *m*/*z* 299.01 and *m*/*z* 241.01. Myricetin is a classic flavonol with a high degree of hydroxylation, which defines its fragmentation pattern.

Core: Flavonol (C2–C3 double bond and a C4 carbonyl).

A-ring: Phloroglucinol-type (1,3,5-trihydroxybenzene)—hydroxyls at C5 and C7.

B-ring: Pyrogallol-type (1,2,3-trihydroxybenzene)—hydroxyls at C3′, C4′, and C5′. This is the key feature that distinguishes it from quercetin (which has a catechol B-ring).

The pyrogallol group is highly susceptible to rearrangement and loss of small neutrals, which produces very characteristic ions.

Loss of C_2_H_2_O_3_: A highly specific rearrangement leading to the loss of a neutral fragment from the B-ring. The mechanism likely involves two hydroxyls and one carbon from the B-ring.

*m*/*z* 317 -> 241 ([M-H-C_2_H_2_O_3_]^−^)

This is a very abundant and characteristic ion for myricetin and other flavonoids with a pyrogallol B-ring. The myricetin was identified in the bibliography in extracts of *Juglans mandshurica* [33], millet grains [34], *Rh. sichotense* [35], *Inula graveolens* [36], *Rh. ungernii* [37], *Solanaceae* [38], *Vitis vinifera* [39], *andean blueberry* [40], *Taraxacum officinale* [41].

The detection of the phytocannabinoid cannabigerorcinic acid in *Rh. adamsii* extracts are extremely noteworthy. Cannabigerorcinic acid was previously identified in extracts of *Rhododendron anthopogon* D. Don [42]. *Rh. anthopogon* (*Ericaceae*) is a medicinal plant that occurs in Swat Valley (a district in the Malakand Division of Khyber Pakhtunkhwa, Pakistan). The structures of two phytocannabinoid derivatives: cannabigerorcinic acid and cannabichromeorcinic acid were elucidated using NMR (nuclear magnetic resonance) spectroscopy and MS.

A highly interesting study has been conducted for the first time on the primary isolation of cannabigerorcinic acid from a fungal source [43]. They studied the bioactive compounds in the fermentation extract of two cultures of *Amylosporus* cf. *graminicola* and *Amylosporus* cf. *campbelii* from Cuba and Zimbabwe and isolated seven previously undescribed secondary metabolites, for which the names amylosporans A–G (1–7) have been proposed. Three additional compounds (8–10), previously unknown from a fungal source, were also characterized for the first time, two of which were assigned the names amylosporans H–I (8–9), while the third was identified as cannabigerorcinic acid (10). The structures of these compounds were determined based on their MS spectra and detailed analysis of NMR spectroscopy data.

**Figure 8:**
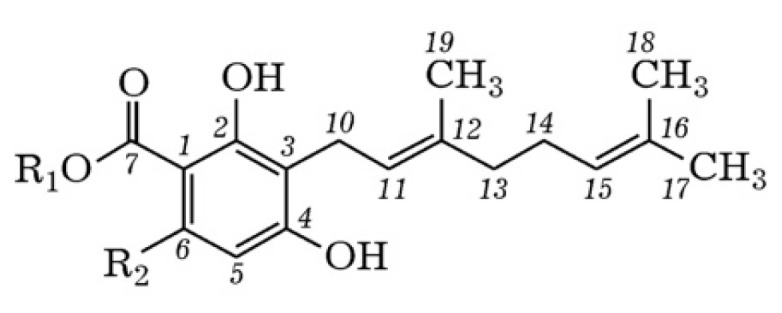
Methyl ester of cannabigerorcinic acid (R_1_ = C^8^H_3_, R_2_ = C^9^H_3_); cannabigerorcinic acid (R_1_=H, R_2_=CH_3_); cannabigerovarinic acid (R_1_ = H, R_2_ = *n*-C_3_H_7_); cannabigerolic acid (R_1_ = H, R_2_ = *n*-C_5_H_11_).

Cannabigerovarinic and cannabigerolic acids are isolated in *Cannabis sativa* (Figure 8), and grifolic acid is found in fungi of the genus *Albatrellus* [44]. Many embryophytes, for example, representatives of the genus *Cannabis* and *Rhododendron*, enter into symbiosis with fungi for a possible pathway of metabolites; therefore, prenylated phenolic compounds are most likely metabolic products of symbionts [45,46].

**Figure 9:**
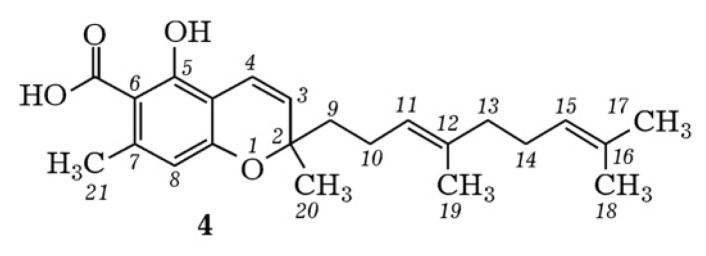
Structural formula of Daurichromenic acid.

Compounds belonging to the class of prenylated phenols, cannabigerorcinic acid methyl ester and daurichromenic acid (Figure 9), are isolated from the natural matrix of *Rh. adamsii* [9].

Daurichromenic acid (DCA), a meroterpenoid composed of orsellinic acid and sesquiterpene moieties, was first isolated from the leaves of *Rhododendron dauricum*. This *Rhododendron* species grows wild in Mongolia, Buryatia, the Russian Far East, northern China, Eastern Siberia, and the Japanese island of Hokkaido [47]. This species of rhododendron is widely used in traditional Chinese medicine as an expectorant and for the treatment of acute and chronic bronchitis [48]. Daurichromenic acid has also been reported to possess anti-inflammatory activity [49] and antibacterial activity against Gram-positive bacteria [50], suggesting that DCA may be a potential resource for the development of precursors that could act as novel therapeutic agents for the treatment of these conditions. DCA has also been noted to exhibit various pharmacological activities, including inducing cell death in cultured cells, but its molecular mechanisms of action remain unclear [51]. Thus, a deeper understanding of its mechanisms of action in human (patho)physiology and its potential therapeutic applications is required. Another potential application for DCA, isolated from *Rh. dauricum*, has also been suggested. Sphingomyelin synthase is a lipid-metabolizing enzyme localized in the cell membrane that plays a key role in cell proliferation, migration, and death [52]. Daurichromenic acid can directly or indirectly influence the activity of sphingomyelin synthase (SMS), regulating cell death and anti-inflammatory activity. Elucidating the mechanism by which DCA interacts with SMS may facilitate the development of new therapeutic agents. Importantly, DCAs non-toxicity makes it a suitable candidate for further development as a novel drug supplement or medicinal product [47].

**Figure 10:**
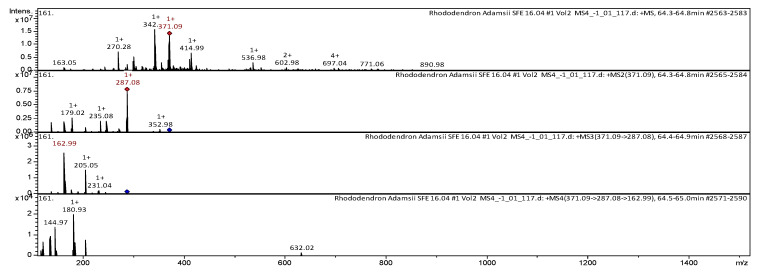
CID-spectrum of daurichromenic acid from CO_2_-extracts of *Rh. adamsii*, *m*/*z* 371.09.

Daurichromenic acid and cannabigerorcinic acid have also been identified by HPLC-MS/MS from *Rh. adamsii* SC-CO_2_ extract. The CID-spectrum in positive ion modes of daurichromenic acid from CO_2_-extracts of *Rh. adamsii* is shown in Figure 10.

**Figure 11:**
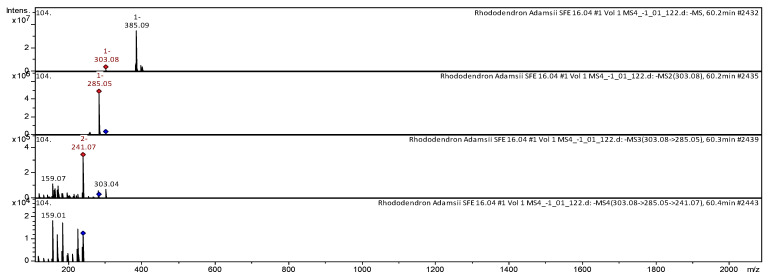
CID-spectrum of cannabigerorcinic acid from CO_2_-extracts of *Rh. adamsii*, *m*/*z* 303.08.

The [M+H]^+^ ion produced one fragment with *m*/*z* 371.09. The fragment ion with *m*/*z* 371.09 yields three daughter ions with *m*/*z* 352.98, *m*/*z* 287.08, and *m*/*z* 235.08. The fragment ion with *m*/*z* 287.08 yields three daughter ions with *m*/*z* 231.04, *m*/*z* 205.05, and *m*/*z* 162.99. Then the fragment ion with *m*/*z* 162.99 yields two daughter ions with *m*/*z* 180.93 and *m*/*z* 144.97. It was identified in the mass spectrometric bibliography in extracts from the leaves of *Rh. dauricum* [47], *Rh. adamsii* [7,8,9]. The CID-spectrum in negative ion modes of cannabigerorcinic acid from CO_2_-extracts of *Rh. adamsii* is shown in Figure 11.

The [M-H]^−^ ion produced one fragment with *m*/*z* 303.08 (Figure 11). The fragment ion with *m*/*z* 303.08 yields one daughter ion with *m*/*z* 285.05. The fragment ion with *m*/*z* 285.05 yields two daughter ions with *m*/*z* 241.07 and *m*/*z* 159.07. Then the fragment ion with *m*/*z* 241.07 yields one daughter ion with *m*/*z* 159.01. It was identified in extracts of *Rh. anthopogon* D. Don [42], *Rh. adamsii* [9], and in the extract of two cultures of *Amylosporus* cf. *graminicola* and *Amylosporus* cf. *campbelii* [43].

## 4. Materials and Methods

### 4.1. Materials

The object of study was purchased samples of *Rh. adamsii* (leaves and stems) from the area near lake Baykal, Russia (53°23′37″ N, 107°45′22″ E). All samples were morphologically authenticated according to the current standard of the Russian Pharmacopeia [53].

### 4.2. Chemicals and Reagents

All reagents used in the study were of analytical grade. HPLC-grade acetonitrile was purchased from Fisher Scientific (Ashford, Kent, UK), and MS-grade formic acid and ethanol (EtOH) were purchased from Sigma-Aldrich (Steinheim, Germany). Ultrapure water was obtained from Siemens (SIEMENS water technologies, Munich, Germany).

### 4.3. Liquid Chromatography

High-performance liquid chromatography was carried out on a Shimadzu LC-20 Prominence HPLC (Shimadzu, Kyoto, Japan) instrument equipped with a UV–vis detector and a C18 silica reverse phase column (4.6 × 150 mm, particle size: 2.7 μm) to perform the separation of these multicomponent mixtures. Mobile-phase eluent A was deionized water containing 0.1% formic acid, and eluent B (CH_3_CN containing 0.1% formic acid). The gradient elution was started at 0–2 min, 0% eluent B, 2–50 min, 0–100% B; control washing: 50–60 min, 100% B. The mobile-phase flow rate and column temperature were maintained at 0.3 mL/min and 30 °C, respectively. A UV–vis detector, the SPD-20A (Shimadzu, Kyoto, Japan), was used at a wavelength of 230 nm. The injection volume was 10 µL. The liquid chromatography Shimadzu LC-20 Prominence HPLC was combined with a mass spectrometric ion trap amaZon SL to identify compounds.

### 4.4. SC-CO_2_ Extraction

SC-CO_2_ extraction was performed using the SFE-500 system (Thar SCF Waters, Milford, CT, USA) supercritical pressure extraction apparatus. System options include: Co-solvent pump (Thar Waters P-50 High Pressure Pump, Milford, CT, USA), for extracting polar samples. CO_2_-flow meter (Siemens, Munich, Germany) to measure the amount of CO_2_ supplied to the system, multiple extraction vessels to extract different sample sizes, or to increase the throughput of the system. The flow rate was 10–25 mL/min for liquid CO_2_ and 1.00 mL/min for co-solvent (C_2_H_5_OH). Extraction samples of 40 g *Rh. adamsii* leaves and stems were used. The extraction time was counted after reaching the pressure with constant CO_2_ flow, and it was 90 min for each sample. This method of SC-CO_2_ extraction of plant matrices was tested by the authors on numerous plant samples, including aboveground and underground parts of the plant [35,54].

### 4.5. Mass Spectrometry

MS analysis was performed on an ion trap amaZon SL (BRUKER DALTONIKS, Bremen, Germany) equipped with an ESI source in negative and positive ion mode. The optimized parameters were obtained as follows: ionization source temperature: 70 °C, gas flow: 4 L/min, nebulizer gas (atomizer): 7.3 psi, capillary voltage: 4500 V, end plate bend voltage: 1500 V, fragmentary: 280 V, collision energy: 60 eV. An ion trap was used in the scan range *m*/*z* 100–1700 for MS and MS/MS. The capture rate was one spectrum/s for MS and two spectrum/s for MS/MS. Data collection was controlled by Windows software for BRUKER DALTONIKS. All experiments were repeated three times. A four-stage ion separation mode (MS/MS mode) was implemented.

## 5. Conclusions

Adaptogenic herbal preparations are considered potential preventative treatments for the symptomatic relief of many chronic diseases. Therefore, expanding the spectrum of known herbal adaptogens is a highly exciting scientific challenge.

Supercritical extraction of *Rh. adamsii* plant matrices using SC-CO_2_ and co-solvent (C2H5OH) as a cosolvent allowed us to obtain samples for analytical study using tandem MS. Forty-nine biologically active compounds were identified in extracts from *Rh. adamsii* leaves and stems. The diversity of isolated biologically active compounds, including flavonoids such as quercetin, kaempferol, dihydroquercetin, myricetin, and others, opens up extensive opportunities for the development of new medicinal products based on extracts of this rhododendron species. It is also worth noting that rare compounds such as the meroterpenoid daurichromenic acid and the phytocannabinoid cannabigerorcinic acid may have been first discovered in *Rh. adamsii*. The pharmacological effects of these compounds require further detailed study.

In this study, we provide a comprehensive conclusion regarding the validity of various ethnopharmacological concepts regarding the beneficial effects of *R. adamsii* extracts on humans as an adaptogen. Further understanding of the mechanisms of action of *R. adamsii* extracts and elucidation of their safety data will require further experiments. However, it is already clear that the perennial plant *R. adamsii* holds promise for both medicinal and therapeutic use.

Future research may offer new therapeutic approaches for the use of both medicinal and cosmetic products based on SC-CO_2_ extracts of *Rh. adamsii*, based on the excellent transdermal penetration of these supercritical extracts.

## Data Availability

The original contributions presented in this study are included in the article. Further inquiries can be directed to the corresponding author.

## Appendix A

**Table A1:** Bioactive substances identified from the SC-CO_2_ extracts of *Rh. adamsii*.

№	Identification	Formula	Calculated Mass	Observed Mass [M-H]^−^	Observed Mass [M+H]^+^	MS/MS Stage 2 Fragmentation	MS/MS Stage 3 Fragmentation	MS/MS Stage 4 Fragmentation	References
	**Group of polyphenols**								
1	**Caffeic acid** [(2E)-3-(3,4-Dihydroxyphenyl)acrylic acid]	**C_9_H_8_O_4_**	180.157		181.08	163.03; 135.11			*Senecio clivicolus* [55]; *Radix polygoni multiflori* [56]; *Lepechinia* [57]; *Rhus coriaria* [58]
2	**Fraxetin**	**C_10_H_8_O_5_**	208.168		209.00	193.12; 165.07	109.2		*Embelia* [59]; *Actinidia* [60]; *Jatropha* [61]; *Artemisia martjanovii* [62]
3	**Kaempferol** [3,5,7-Trihydroxy-2-(4-hydro- xyphenyl)-4H-chromen-4-one]	**C_15_H_10_O_6_**	286.236		287.00	286.24; 204.96; 163.02	181.02	162.88	*Rh. adamsii* [63,64]; *Rhus coriaria* [58]; *Lonicera japonica* [65]; *Ribes meyeri* [66]
4	**Rhamnocitrin**	**C_6_H_12_O_6_**	300.263		301.02	163.03; 283.02	283.02 181.00		*Mentha* [29]; *Astragali radix* [30]; *Lonicera caerulea* [31]; *Phyllanthus urinaria L.* [32]
5	**Ellagic acid** [Benzoaric acid; Elagostasine; Lagistase; Eleagic acid]	**C_14_H_6_O_8_**	302.193		303.13	285.06	257.03	201.02	*Cicer arietinum* [67]; *Punica granatum* [68]; *Juglans regia* [69]; *Juglans mandshurica* [33]; *Myrtle* [70]; *Rhus coriaria* [58]
6	**Quercetin** [2-(3,4-Dihydroxyphenyl)-3,5,7-trihy- droxy-4H-chromen-4-one]	**C_15_H_10_O_7_**	302.235	301.02	303.08	285.01; 163.02	180.97; 145.00	162.98	*Ribes meyeri* [66]; *Ribes dikuscha; Ribes triste* [71]; *Capsicum annuum* [72]; *Propolis* [73]
7	**Hesperitin** [Hesperetin]	**C_16_H_14_O_6_**	302.279	301.05		283.00; 217.02; 167.03	239.02; 149.03		*Vitis vinifera* [39]; *Mentha* [74]; *Rosmarinus officinalis* [75]
8	**Dihydroquercetin** [Taxifolin; Taxifoliol]	**C_15_H_12_O_7_**	304.251	303.09		285.04	266.96; 241.09; 215.05; 135.05	171.02	millet grains [34]; *Ribes triste* [71]; *Potentilla freyniana* [76]; *Thymus vulgaris; Oregano* [77]
9	**Isorhamnetin** [Isorhamnetol; Quercetin 3′-Methyl ether]	**C_16_H_12_O_7_**	316.262		317.07	299.04; 177.02	146.98		*Phoenix dactylifera* [78]; *Cyperus laevigatus* [79]; *Spondias purpurea* [80]
10	**Myricetin** [3,5,7-Trihydroxy-2-(3,4,5-Trihydroxyphenyl)-4H-Chromen-4-One]	**C_15_H_10_O_8_**	318.235	317.08		299.01; 241.01	240.06; 197.09	238.99; 197.04	*Juglans mandshurica* [33]; millet grains [34]; *Rh. sichotense* [35]; *Inula graveolens* [36]; *Rh. ungernii* [37]; *Solanaceae* [38]; *Vitis vinifera* [39]; *Andean blueberry* [40]; *Taraxacum officinale* [41]
11	**Quercetagetin**	**C_15_H_10_O_8_**	318.235	317.08		299.05; 203.11	255.13		*F. herrerae* [81]
12	**Fertaric acid** [2-O-(4-Hydroxy-3-Methoxy-trans-cinnamoyl)tartaric acid]	**C_14_H_14_O_9_**	326.256		327.08	271.01; 177.06; 217.03	149.10		*Vitis vinifera* [39]; *Melissa officinalis* [82]; *Salvia; Mint; Basilic* [77]
13	**Esculin** [Aesculin; Esculoside; Polichrome]	**C_15_H_16_O_9_**	340.282		341.09	281.01; 217.11; 151.06	174.96		*Rh. sichotense* [35]; *Artemisia annua* [83]; *Stevia rebaudiana* [84]; *Actinidia chinensis* [60]
14	**Caffeic acid-O-hexoside** [Caffeoyl-O-hexoside]	**C_15_H_18_O_9_**	342.298		343.09	243.01; 163.00			*Myrtle* [70]; *Cranberry* [85]; *Andean blueberry* [40]; *Cherimoya, papaya* [86]; Rapeseed petals [87]; *Phoenix dactylifera* [78]
15	**Sesamin** [Fagarol; Sezamin; Asarinin]	**C_20_H_18_O_6_**	354.353		355.10	337.02; 231.02; 164.26			*Eleuterococcus* [88]; *Lignans* [89]; *Petroselenium crispum* [77]
16	**Fraxin** (Fraxetin-8-O-glucoside)	**C_16_H_18_O_10_**	370.308		371.08	338.99	320.96; 177.03	224.96	*Rh. sichotense* [35]; *Actinidia chinensis* [60]; *Solanum tuberosum* [90]
17	**Fraxetin-7-O-beta-glucuronide**	**C_16_H_16_O_11_**	384.291	383.09		365.09; 190.96	266.97; 215.02	170.97	*Rh. sichotense* [35]; rat plasma [91]
18	**Cyanidin-3-alpfa-L-arabinoside**	**C_20_H_19_O_10_**	419.358	418.51		399.05; 319.02; 194.99	381.068 162.02	337.02; 253.08	*Chokeberry* [92]
19	**Avicularin** (Quercetin 3-Alpha-L-Arabinofuranoside; Avicularoside)	**C_20_H_18_O_11_**	434.350	433.09		415.07; 335.01; 176.98	397.06; 190.99	353.07; 253.99	*Rh. sichotense* [35]; *Ribes meyeri* [66]; *Cranberry* [85]; *Juglans mandshurica* [33]; *Loropetalum chinense* [93]
20	**Taxifolin-O-pentoside** [Dihydroquercetin pentoside]	**C_20_H_20_O_11_**	436.371	435.16		416.54; 300.99; 231.01	397.02; 205.96	361.11; 283.02; 188.80	*Rh. sichotense* [35]; *millet grains* [34]; *Rosa davurica* [94]; *Chilean currants* [95]
21	**Quercitrin** [Quercetin 3 L- Rhamnoside; Quercetrin]	**C_21_H_20_O_11_**	448.376		448.89	370.95; 282.93	352.95; 176.98	334.90; 222.92; 176.97	*Propolis* [73]; *Embelia* [59]; *Euphorbia hirta* [96]
	**Other chemical groups**								
22	**Tetrahydroxypentanoic acid**	**C_5_H_10_O_6_**	166.129	165.06		147.01			*Cyperus laevigatus* [79]
23	**Glucaric acid** [D-Glucaric acid; Saccharic acid; D-Glutarate]	**C_6_H_10_O_8_**	210.139		211.09	192.12	175.06; 136.12		*Soybean* [97]; *Cherimoya, Papaya* [86]
24	**Stearidonic acid** [6,9,12,15-Octadecatetraenoic acid; Moroctic acid]	**C_18_H_28_O_2_**	276.414		277.09	275.04; 207.05	256.99		*Salviae Miltiorrhizae* [98]; *G. linguiforme* [81]; *Rhus coriaria* [58]; *Lonicera caerulea* [99]; *Jatropha* [61]
25	**Linoleic acid** [Linolic acid; Telfairic acid]	**C_18_H_32_O_2_**	280.445		281.99	264.94; 152.01; 163.00	180.95; 135.06	162.99	*Zostera marina* [100]; *Jatropha* [61]
26	**3,4-Dihydroxyestran-17-one**	**C_18_H_28_O_3_**	292.413		293.05	274.98; 146.97	256.99; 162.98	201.03	*Juglans mandshurica* [63]
27	**Nonadecanoic acid** [N-Nonadecanoic acid]	**C_19_H_38_O_2_**	298.503		300.09	243.04	201.02		*Rh. adamsii* [7,8,9]
28	**3-Hydroxy-9,10-dimethoxypterocarpan**	**C_17_H_16_O_5_**	300.306		300.09	243.04	201.02		*Huolisu Oral Liquid* [101]; *Radix astragali* [30]; *Chinese herbal formula Jian-Pi-Yi-Shen pill* [102]
29	**Cannabigerorcinic acid** [Cannabigerorcinolic acid; Cannabiorcogerolic acid]	**C_18_H_24_O_4_**	304.380	303.08		285.05	241.07; 159.07	159.01	*Rh. adamsii* [7,8,9]
30	**8-Demethyleucalyptin** [5-Hydroxy-4′,7-dimetoxy-6-methylflavone; Pabalate; Sodium salicylate]	**C_18_H_16_O_5_**	312.316	311.14		182.99			*l. palustre* [103]
31	**Arachic acid** [Arachidic acid; eicosanoic acid]	**C_20_H_40_O_2_**	312.530	311.14		299.01; 287.15	256.01; 239.21;		*F. herrerae; C. edulis* [81]; *Cyperus laevigatus* [79]
32	**4-O-p-coumaroyl shiikimic acid**	**C_16_H_16_O_7_**	320.294	319.10		275.08	257.10; 217.13	257.08	*Andean blueberry* [40]; *Lonicera caerulea* [31]
33	**Bilobalide** [(-)-Bilobalide]	**C_15_H_18_O_8_**	326.299	325.11		182.99			*Ginkgo biloba* [104]; *Malus toringoides* [105]; *Xindxiong injection* [106]
34	**Gingerenone C**	**C_20_H_22_O_4_**	326.386		327.15	203.06; 137.05	175.03		*Ventilago denticulata* [107]
35	**Mukurozidiol** [Byakangellicin]	**C_17_H_18_O_7_**	334.321		335.04	303.06; 195.01	284.99; 163.00	135.14	*Ventilago denticulata* [107]
36	**4,17-Dimethoxy-2-oxatricyclo [13.2.2.1-3,7-]-icosa-1(17),3(20),4,6,15,18-hexaen-10-one**	**C_21_H_24_O_4_**	340.412		341.09	281.06; 217.04; 243.06	137.05		*Juglans mandshurica* [33]
37	**Behenic acid** [Docosanoic acid]	**C_22_H_44_O_2_**	340.583		341.05	323.10; 243.11; 177.04	159.05		*Rh. adamsii* [7,8,9]; *Pinus sylvestris* [108]
38	**Isochlorogenic acid**	**C_16_H_18_O_9_**	354.309		355.03	323.00; 227.05	296.96; 172.96		*Actinidia chinensis* [60]
39	**Tricosanoic acid** [N-Tricosanoic acid]	**C_23_H_46_O_2_**	354.610		355.08	322.96; 163.00	180.96	162.96	*Rh. adamsii* [7,8,9]
40	**Lignoceric acid** [Tetracosanoic acid]	**C_24_H_48_O_2_**	368.636	367.12	369.08	351.08; 285.02; 218.92; 162.98	163.02	144.97	*Rh. adamsii* [7,8,9]
41	**Daurichromenic acid**	**C_23_H_30_O_4_**	370.481		371.09	352.98; 287.08; 235.08; 179.02	231.04; 205.05; 162.99	180.93; 144.97	*Rh. adamsii* [7,8,9]; *Rh. sichotense* [35]
42	**Pentacosanoic acid** [N-Pentacosanoic acid]	**C_25_H_50_O_2_**	382.663		383.07	351.04; 287.99	229.04	211.03	*Rh. adamsii* [7,8,9]; *Rh. sichotense* [35]
43	**Desmosterol** [24-Dehydrocholesterol; 3beta-Cholesa-5,24-Dien-3-Ol]	**C_22_H_24_O_4_**	384.422		385.06	367.02; 300.92;	282.94	162.89	*A. cordifolia* [81]
44	**3-(4,5-Dihydroxy-2-methyl-9,10-dioxo-9,10-dihydro-1-anthracenyl)-2,6-dihydroxy-1-benzoic acid**	**C_22_H_12_O_8_**	404.326		405.03	372.94; 244.95	354.95; 229.93	336.95; 283.52; 216 93	*Juglans mandshurica* [33]
45	**Beta-Sitosterin** [Beta-Sitosterol]	**C_29_H_50_O**	414.706		415.04	384.02	369.01	338.00	*Rh. sichotense* [35]; *F. herrerae; C. edulis* [81]; *Pinus sylvestris* [108]
46	**Lupa-2,20(29)-dien-28-ol**	**C_30_H_48_O**	424.701		425.01	406.95; 202.91	389.01; 298.98	265.86; 203.86	*Juglans mandshurica* [33]
47	**Lupeol** [Fagarasterol; Clerodol; Monogynol B; Lupenol]	**C_30_H_50_O**	426.717		427.04	409.01; 202.99	389.02; 247.99	370.96; 264.80	*Salvia* [109]; *Juglans mandshurica* [33]
48	**Uvaol**	**C_30_H_50_O_2_**	442.716		443.22	425.01; 233.07	407.02; 325.01	388.99; 231.11	*Rh. sichotense* [35]; *F. herrerae; C. edulis* [81]; *Olive leaves* [110]
49	**3-Oxoolean-12-en-29-oic acid**	**C_30_H_46_O_3_**	454.684		455.05	409.00	390.98; 256.93	250.80; 212.08	*Juglans mandshurica* [33]
